# Transcriptome analysis of microRNA156 overexpression alfalfa roots under drought stress

**DOI:** 10.1038/s41598-018-27088-8

**Published:** 2018-06-19

**Authors:** Muhammad Arshad, Margaret Y. Gruber, Abdelali Hannoufa

**Affiliations:** 1 Agriculture and Agri-Food Canada, 1391 Sandford Street, London, Ontario, N5V 4T3 Canada; 2 Centre of Agricultural Biochemistry and Biotechnology, University of Agriculture, Faisalabad, Pakistan; 3 Agriculture and Agri-Food Canada, 107 Science Place, Saskatoon, SK S7N 0X2 Canada

## Abstract

Drought is one of the major abiotic stresses that negatively impact alfalfa growth and productivity. The role of microRNA156 (miR156) in drought has been demonstrated in plants. To date, there are no published studies investigating the role of miR156 in regulating global gene expression in alfalfa under drought. In our study, alfalfa genotypes overexpressing miR156 (miR156OE) exhibited reduced water loss, and enhanced root growth under drought. Our RNA-seq data showed that in response to drought, a total of 415 genes were upregulated and 169 genes were downregulated specifically in miR156OE genotypes. Genotypic comparison revealed that 285 genes were upregulated and 253 genes were downregulated in miR156OE genotypes relative to corresponding WT under drought. Gene Ontology enrichment analysis revealed that the number of differentially expressed genes belonging to biological process, molecular function and cell component functional groups was decreased in miR156OE genotypes under drought. Furthermore, RNA-Seq data showed downregulation of a gene encoding WD40 repeat in a miR156-specific manner. 5′ RACE experiments verified cleavage of *WD40-2* transcript under drought. Moreover, alfalfa plants overexpressing *WD40-2* showed drought sensitive, whereas those with silenced *WD40-2* exhibited drought tolerant phenotypes. These findings suggest that miR156 improves drought tolerance in alfalfa by targeting *WD40-2*.

## Introduction

Crop losses caused by extreme environmental conditions have risen steadily over the past decades^1^. Climate change models predict more frequent incidence of drought and extreme temperature in the near future^2–7^. An increased demand for water consumption by plants suggests the need for stress resilient crop genotypes to ensure sustainable food production^8^.

Alfalfa (*Medicago sativa*) is the most widely cultivated forage legume crop in the world^9^. In addition to its primary use as a forage for livestock feed, alfalfa enhances soil fertility by fixing atmospheric nitrogen^10^. Furthermore, alfalfa is contemplated for use as a feedstock for biofuel production, a practice that would contribute to a cleaner environment^11^. However, drought negatively affects alfalfa growth and biomass yield^12^.

Genome-wide transcriptome analysis has emerged as a powerful tool to discover genes that regulate various traits in plants. A transcriptomics study of root-knot nematode resistant and susceptible alfalfa genotypes revealed several differentially expressed genes common to both genotypes, as well as genes unique to individual genotypes^13^. Leaf transcriptome analysis of these nematode-related genotypes also led to the identification of candidate genes related to fall dormancy^14^. Transcriptome analysis of *Medicago truncatula* has been conducted in response to salt stress^15,16^. Moreover, the full sequence of *M*. *truncula* genome is available to use as a reference to discover alfalfa (*Medicago sativa*) genes involved in various traits such as abiotic stress^17^. The *M*. *truncatula* genome has been used to annotate transcriptome profiles of alfalfa under salinity stress^13,18^^,^. Although alfalfa and *M*. *trunctula* share a high degree of sequence similarity, genetic diversity between the two still exists^19^. Hence, genetic information obtained from the *M*. *truncatula* genome may not always be applicable to alfalfa. This is apparent from the large number of unannotated genes in a recently published salt resistant study on alfalfa breeding populations^20^.

MicroRNAs are eukaryotic gene regulators that repress gene expression by inducing transcript cleavage or translation repression^21^. Several studies have been undertaken to discover the role of microRNAs in improving abiotic stresses in numerous plants^22–24^.

MicroRNA156 (miR156) and its *SPL* target genes play crucial roles in regulating different aspects of growth, development, and flowering in many plant species^25–31^. Apart from *SPL* genes, miR156 is known to regulate non-conserved *WD*40 genes, which function in signal transduction, transcription regulation, and apoptosis in eukaryotes^32^. Naya *et al*.^32^ showed that a specific isoform of miR156 cleaves non-conserved *WD*40 targets in *M*. *truncatula* root apices. Although *WD40* regulatory genes are known mainly for their role in anthocyanin biosynthesis and trichome development in plants^33–36^, there are other studies that link *WD40* to nodule and cell wall formation^37,38^, and response to hormones, light and abiotic stress^39^.

More recently, we showed a positive role of miR156 in drought and salinity stress responses of alfalfa^12,40^. Despite a series of miR156-related studies in various plant species, there has been no reported transcriptome analysis on miR156OE alfalfa roots under drought stress. A root transcriptome analysis of contrasting alfalfa genotypes under these conditions could provide an insight into the underlying molecular mechanisms that control this trait in alfalfa. Hence, we conducted RNA sequencing (RNA-Seq) analysis on two miR156OE alfalfa genotypes (A8 and A16b) generated in a previous study^25^, after exposure to drought. Previously, these two genotypes showed improved drought tolerance and root biomass under drought^12^. In the current study, these genotypes exhibited increased root length and reduced water loss. The present analysis identified differentially expressed genes in alfalfa roots and investigated the role of a miR156-targeted *WD40-2* gene in modulating drought responses in alfalfa.

## Results

### MiR156 promotes root growth and reduces water loss under drought conditions

**Figure 1:**
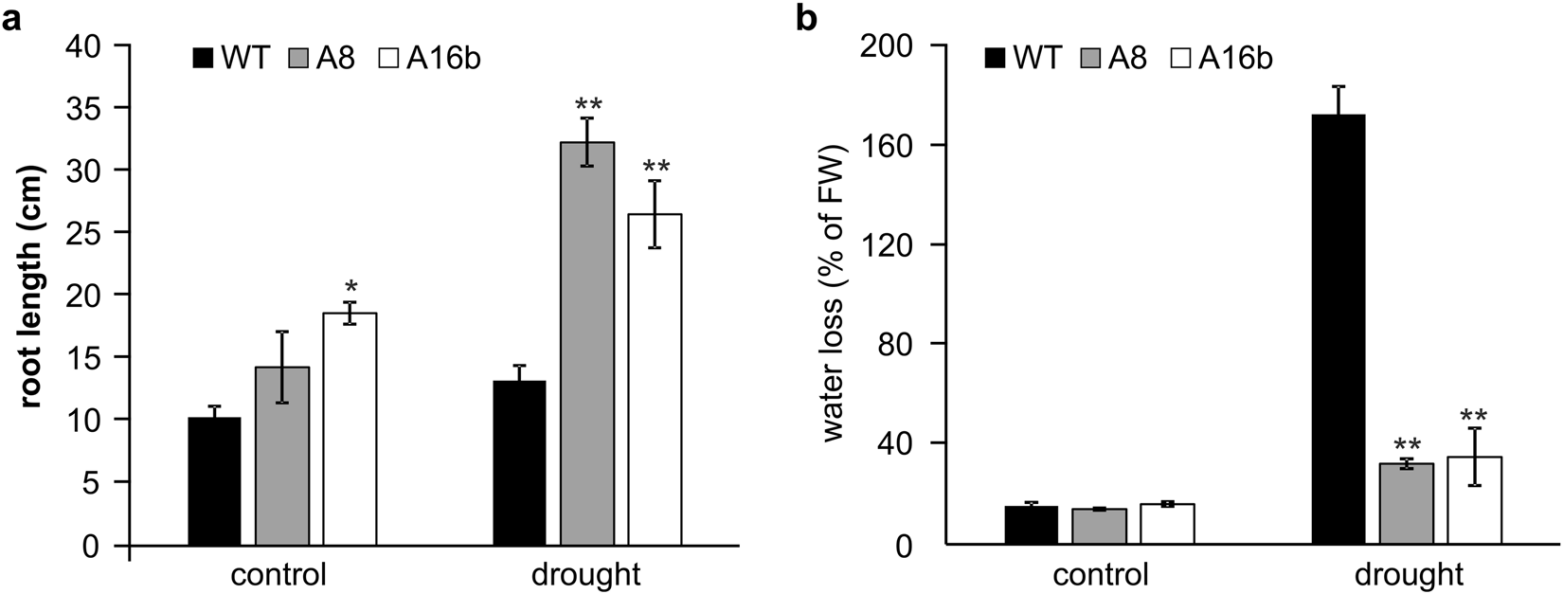
Physiological response of WT and miR156OE genotypes to drought. (**a**) Root length, (**b**) water loss, measurements in drought stressed and well-watered control WT and miR156OE plants at day 13. An asterisk (*) shows statistical significance at p < 0.05 while double asterisk (**) indicates significance at p < 0.01 where n = 3–8 (ANOVA).

We measured root length of WT and miR156OE plants under control and drought stress. Under non-stress control conditions, A16b had longer roots than WT, whereas no statistical differences were observed between A8 and WT under non-stress conditions (Fig. 1a). Upon exposure to drought, we observed even more pronounced differences in root length between WT and miR156OE plants. Both A8 and A16b genotypes showed longer roots than WT (Fig. 1a). Drought-induced increase in root length ranged from 42% (A16b) to 130% (A8) in miR156OE plants after 13 days of drought stress, whereas only a 12% increase was recorded in WT during this period (Fig. 1a). This experiment provides evidence that miR156 overexpression enhances root growth under drought conditions.

In a water loss assay, we did not observe differences between miR156OE and WT under control conditions, whereas upon withholding water for 12 days, water loss in miR156OE plants was reduced (~137%) compared to WT (Fig. 1b). This provided additional evidence that miR156 plays a role in water conservation during drought stress. In addition to the above results, A16b and A8 exhibited other drought tolerance traits which were reported in our previous study^12^.

### *De novo* assembly of alfalfa root transcriptome

The samples of WT and the two most prominent miR156OE genotypes A16b and A8^25^ were selected after drought stress for next generation sequencing to identify differentially expressed genes (DEGs). A total of 755,528,618 short reads were generated from cDNA libraries of non-stressed and drought stressed roots of WT, A8 and A16b (Supplementary Table 1). Poor quality reads such as adapters, short reads and unpaired reads were filtered out and high quality reads were obtained (Supplementary file S1).

**Figure 2:**
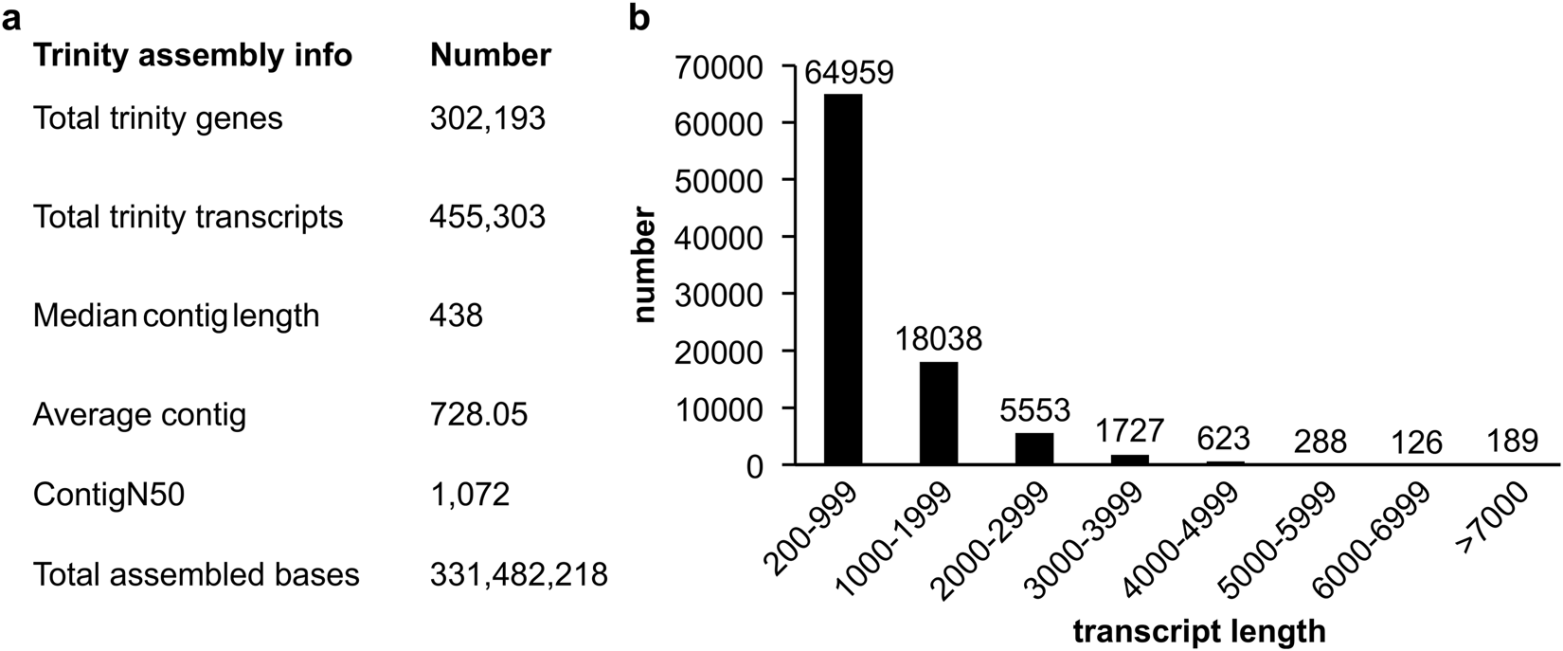
Summarized statistics of *de novo* assembly of *Medicago sativa* transcriptome using the Trinity program. (**a**) Summary of *de novo* assembly of *M*. *sativa* root transcriptome, (**b**) assembled transcript length distribution.

Due to the absence of alfalfa genome sequences, we performed *de novo* assembly on alfalfa root transcriptome using Trinity assembly program^41^. From *de novo* assembly, a total of 455,303 transcripts were obtained with an average length of 728.05 bp. We recorded a N50 value of 1072 bp (Fig. 2a, Supplementary file S2), which was higher than what was previously reported (Postnikova *et al*.^13^). The *de novo* assembly contained a total of 302,193 Trinity genes (Fig. 2a), of which 64,959 genes were between 200–999 bp long and constituted the majority of the genes (Fig. 2b).

### Drought-related DEGs

**Figure 3:**
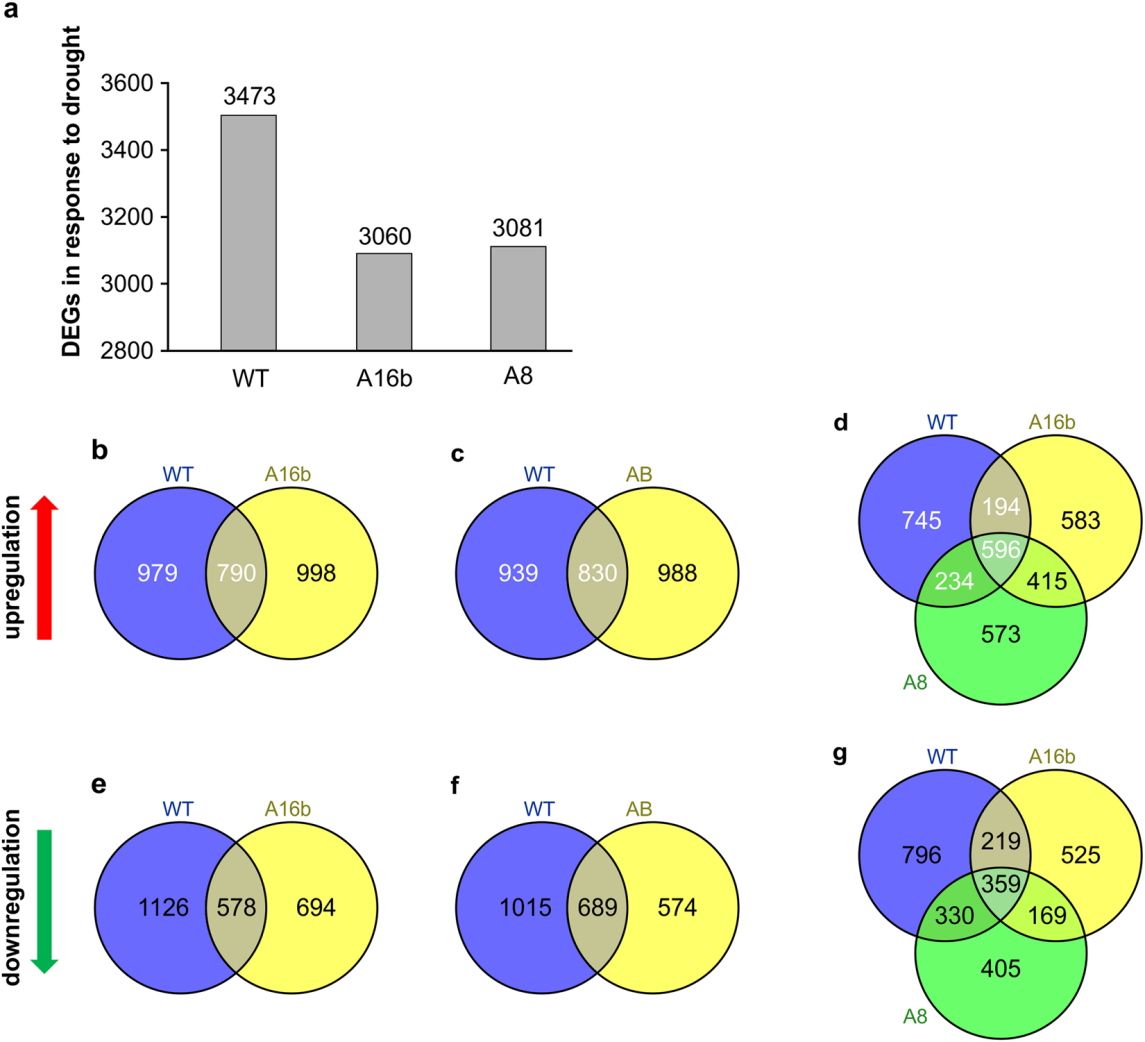
Comparison of significant DEGs found in WT and two miR156OE genotypes in response to drought stress. Venn diagram shows statistically significant DEGs in (**a**) total, (**b**–**d**) upregulated, and (**e**–**g**) downregulated, in WT, A8 and A16b under drought stress conditions relative to corresponding well-watered control plants.

We carried out root transcriptome analysis in drought stressed plants to identify DEGs that may contribute to drought stress responses. We compared expression between non-stressed control and drought stressed roots of WT, A8 and A16b genotypes. To that affect, we observed a reduced number of total DEGs in both miR156OE genotypes (A8, A16b) compared to WT under drought (Fig. 3a, Supplementary files S3, S4, S5). A total of 998 and 988 unique DEGs were significantly upregulated in response to drought specifically in either A16b or A8, respectively, and not in WT (Fig. 3b,c, Supplementary files S3, S4, S5), with 415 DEGs being common genes upregulated both in A16b and A8 (Fig. 3d, Supplementary files S3, S4, S5). Similarly, we found 694 DEGs that were downregulated in response to drought in A16b, 574 DEGs in A8, but none of the genes were silenced in WT (Fig. 3,f, Supplementary files S3, S4, S5). Furthermore, we observed a downregulation of 169 DEGs in response to drought in both miR156OE genotypes (Fig. 3g, Supplementary files S3, S4, S5), indicating these DEGs may be regulated by miR156 in response to drought.

### Genotypic differences in DEGs

**Figure 4:**
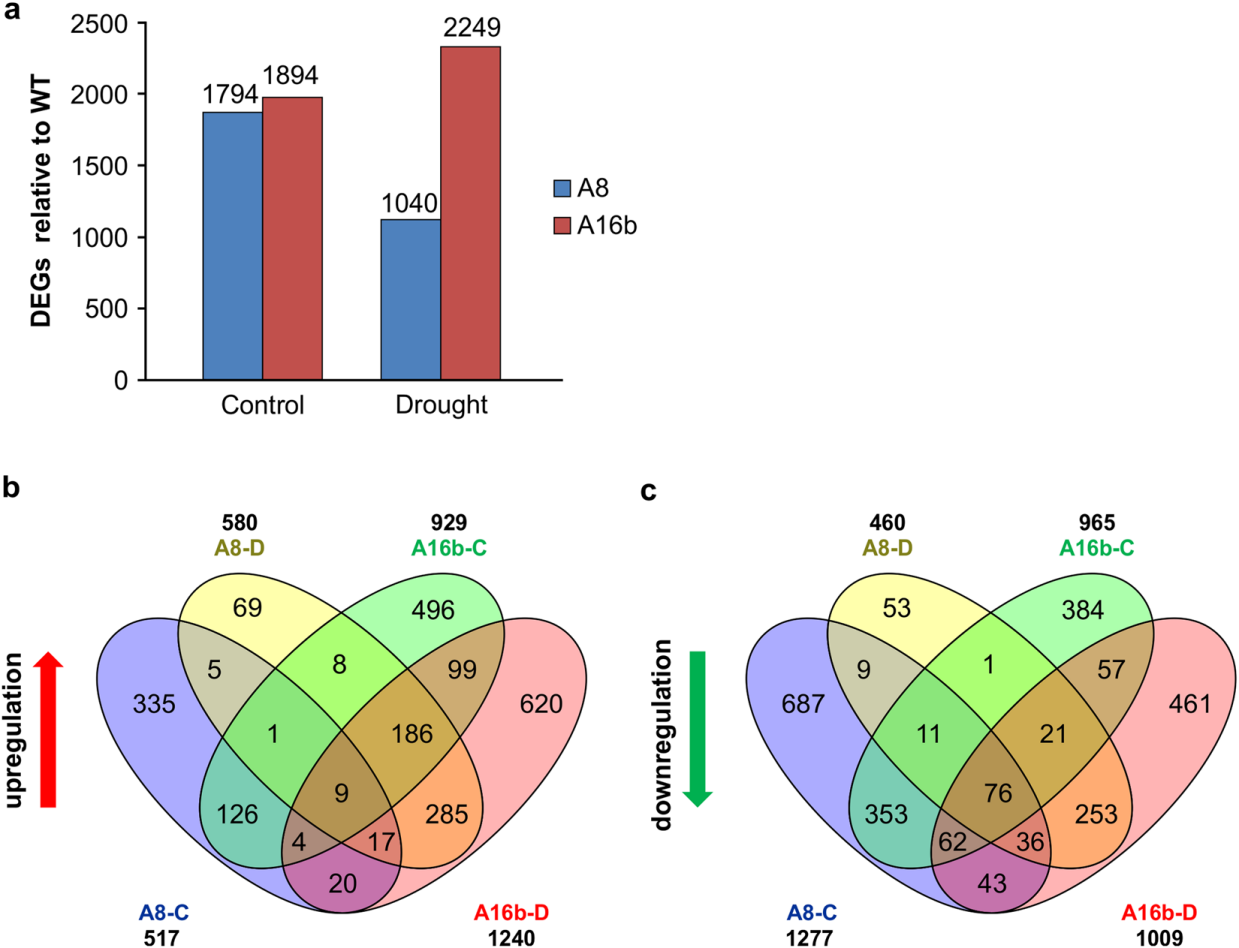
Comparison of significant DEGs found in two miR156OE genotypes relative to corresponding WT. Venn diagram shows statistically significant DEGs (**a**) total, (**b**) upregulated and (**c**) downregulated in A8 and A16b under well-watered control (A8-C, A16b-C) or drought stress (A8-D, A16b-D) conditions relative to corresponding WT.

A total of 1794 and 1894 DEGs were found in A8 and A16b, respectively, relative to WT under non-stress conditions. Furthermore, we observed contrasting effects of drought on a number of DEGs in these two miR156OE genotypes where drought increased the number of DEGs to 2249 in A16b but reduced it to 1040 in A8 (Fig. 4a; Supplementary files S6, S7, S8 and S9). Of the total DEGs in A8 under control conditions, 517 genes were upregulated and 1277 were downregulated relative to WT (Fig. 4b,c, Supplementary file S6). Number of DEGs in A8 was reduced under drought stress when 580 genes were upregulated and 460 were downregulated relative to WT (Fig. 4b,c, Supplementary file S8). On the other hand in A16b, a total of 929 genes were upregulated and 965 were downregulated under control conditions relative to the corresponding WT (Fig. 4b,c, Supplementary file S7). Under drought stress, number of DEGs was increased in A16b with 1240 genes showing upregulation and 1009 downregulation relative to the corresponding WT (Fig. 4b,c, Supplementary file 9). We detected 538 differentially expressed genes common between A8 and A16b under drought stress conditions (Fig. 4b,c), indicating these genes may be specifically regulated by miR156 under drought conditions.

### Gene ontology (GO) enrichment analysis

**Figure 5:**
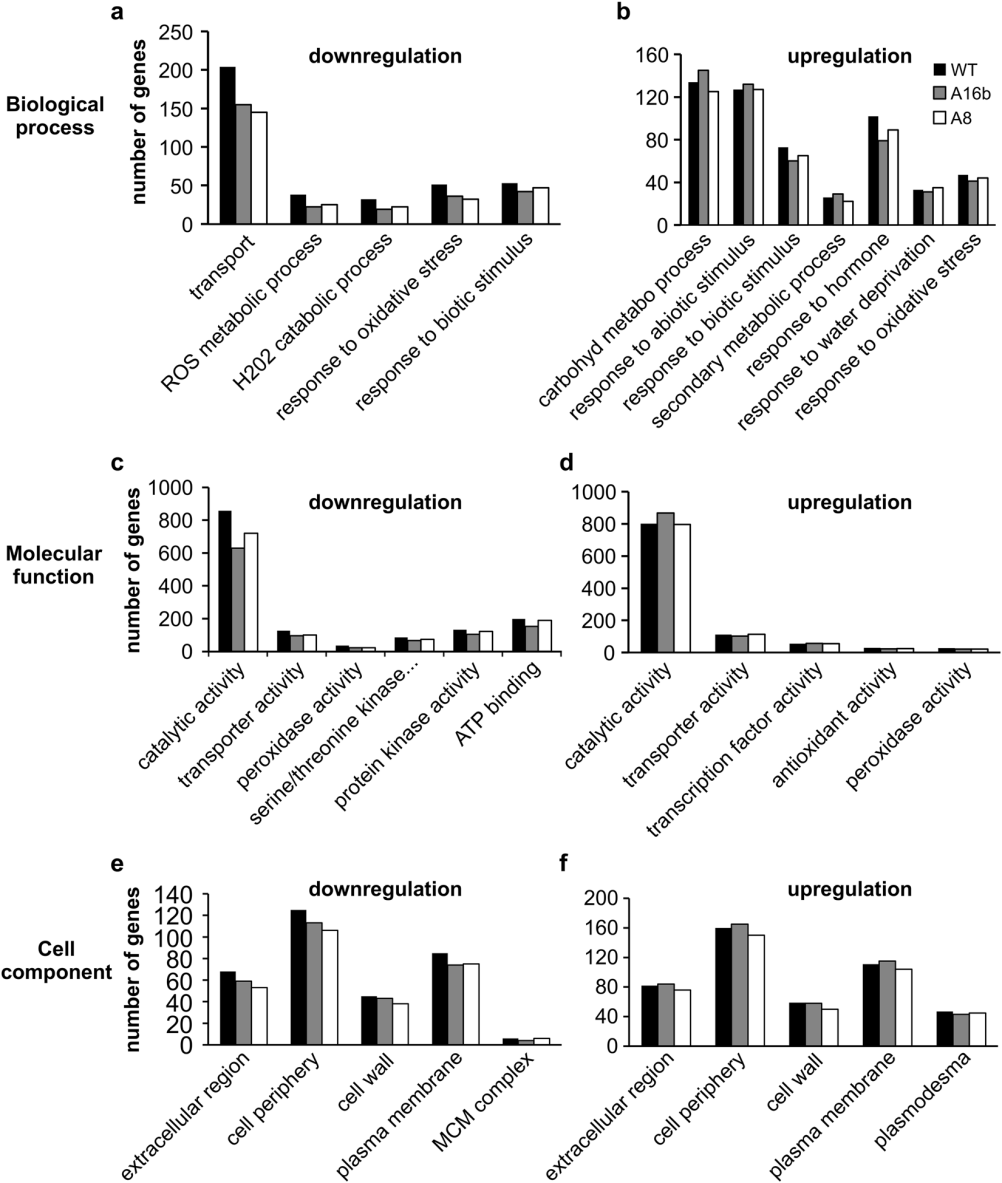
Distribution of gene ontology (GO) categories involved in drought stress responses among three GO domains. Left panel (**a**,**c**,**e**) indicates number of downregulated whereas right panel (**b**,**d**,**f**) shows upregulated DEGs in WT and miR156OE genotypes in response to drought stress.

GO analysis revealed that drought reduced the number of DEGs belonging to various functional groups in miR156OE roots compared to WT (Fig. 5). In biological process, we found a reduced number of DEGs that were downregulated in miR156OE genotypes compared to WT (Fig. 5a), whereas either one or both miR156OE genotypes showed a small increase in the number of DEGs that were upregulated in carbohydrate metabolic process, response to abiotic stimulus, secondary metabolite process and response to water deprivation (Fig. 5b). Similar results were observed in the molecular function category where the number of downregulated genes was reduced in miR156OE roots compared to WT (Fig. 5c). A small increase in the number of upregulated genes was observed in one or both miR156OE genotypes in the functional sub-categories of catalytic activity, transporter activity and transcription factor activity (Fig. 5d). Consistently, a decreased number of DEGs was recorded in the cellular component category in miR156OE genotypes compared to WT (Fig. 5e), but an increased number of upregulated genes was observed in one or both miR156OE genotypes in response to drought in this category. These latter sub-categories include extracellular region, cell periphery and plasma membrane (Fig. 5f).

### RNA-Seq data validation by quantitative real time PCR

We validated RNA-Seq data by randomly selecting 27 genes that included upregulated, downregulated, and unchanged expression in response to drought stress and/or miR156. These genes were tested for expression pattern consistency using quantitative real time PCR (qRT-PCR). The expression levels of the selected genes in response to drought stress (relative to non-stress controls) was compared with RNA-Seq data and presented in Supplementary Table 2. A strong correlation existed between the two expression data sets showing Pearson correlation coefficient r = 0.82 (Supplementary file S10). Under different drought stress levels and genotypes, NGS and RT-qPCR data showed similar trends, providing strong confidence in our RNA-seq data.

### Identification of drought and miR156 responsive transcription factors

Using the Plant Transcription Factor Database (http://planttfdb.cbi.pku.edu.cn/), we identified a number of transcription factors that were significantly upregulated or downregulated in response to drought and miR156. Among major classes of transcription factors, number of up-regulated C2H2 was increased, whereas TCP was decreased in miR156OE genotypes under drought stress conditions (Supplementary Table 3). Genotypically, drought affected more bZIP TFs in A16b than A8. In addition, we observed a decreased number of WRKY genes that were downregulated in miR156OE genotypes under drought relative to non-stress conditions (Supplementary Table 3). Moreover, number of downregulated SBP-box transcription factor was increased in miR156OE genotypes, whereas WD40 repeat was similar under drought stress conditions (Supplementary Table 3).

### MiR156 regulation of *WD40 genes* under drought

**Figure 6:**
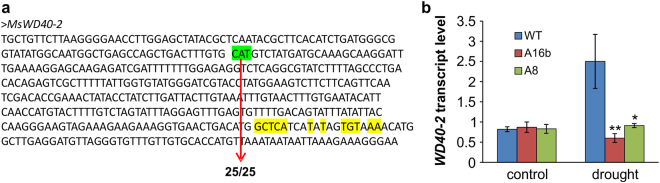
*WD40-2* cleavage in root of miR156OE genotypes. (**a**) Validation of *WD40-2* cleavages by 5′ RACE in transgenic alfalfa overexpressing miR156. The predicted *miR156* target sequences are highlighted in yellow. The *WD40-2* sequences are shown and cleavage sites are highlighted in green. The denominator refers to the number of clones sequenced whereas the numerator represents the number of clones cleaved. (**b**) qRT-PCR analysis of *WD40-2* expression in roots of WT and miR156OE genotypes under well-watered control and drought stress conditions.

Our RNA-seq analysis showed that expression of *WD40-2* (*Medtr2g028050*) was reduced in miR156OE genotypes under drought stress (Supplementary files S8, S9). To investigate whether *miR156* targets the *WD40-2*, we identified putative *miR156* recognition sites using sequence alignment, and carried out 5′-RACE experiments^25^ to analyze cleavage of the *WD40-2* transcript. Transcript cleavage was detected outside of the predicted miR156 target sites in all 25 sequenced clones (Fig. 6a). On further investigation with qRT-PCR, we observed downregulation of *WD40-2* in miR156OE genotypes, but only under drought stress, as non-stressed control plants did not show a difference in *WD40-2* transcript level (Fig. 6b).

### Misexpression of *WD40-2* affects growth and drought tolerance in alfalfa

Further, we generated overexpression and RNA interference (RNAi) genotypes. This included four transgenic genotypes overexpressing *WD40-2* (WD-1OE, WD-4OE, WD-6OE and WD-7OE) (Supplementary file S11), and four RNAi plants with reduced *WD40-2* levels (WD-1R, WD-3R, WD-17R, WD-18R) (Supplementary file S11). These *WD40-2* overexpression, RNAi and WT plants were then tested for tolerance to drought stress.

**Figure 7:**
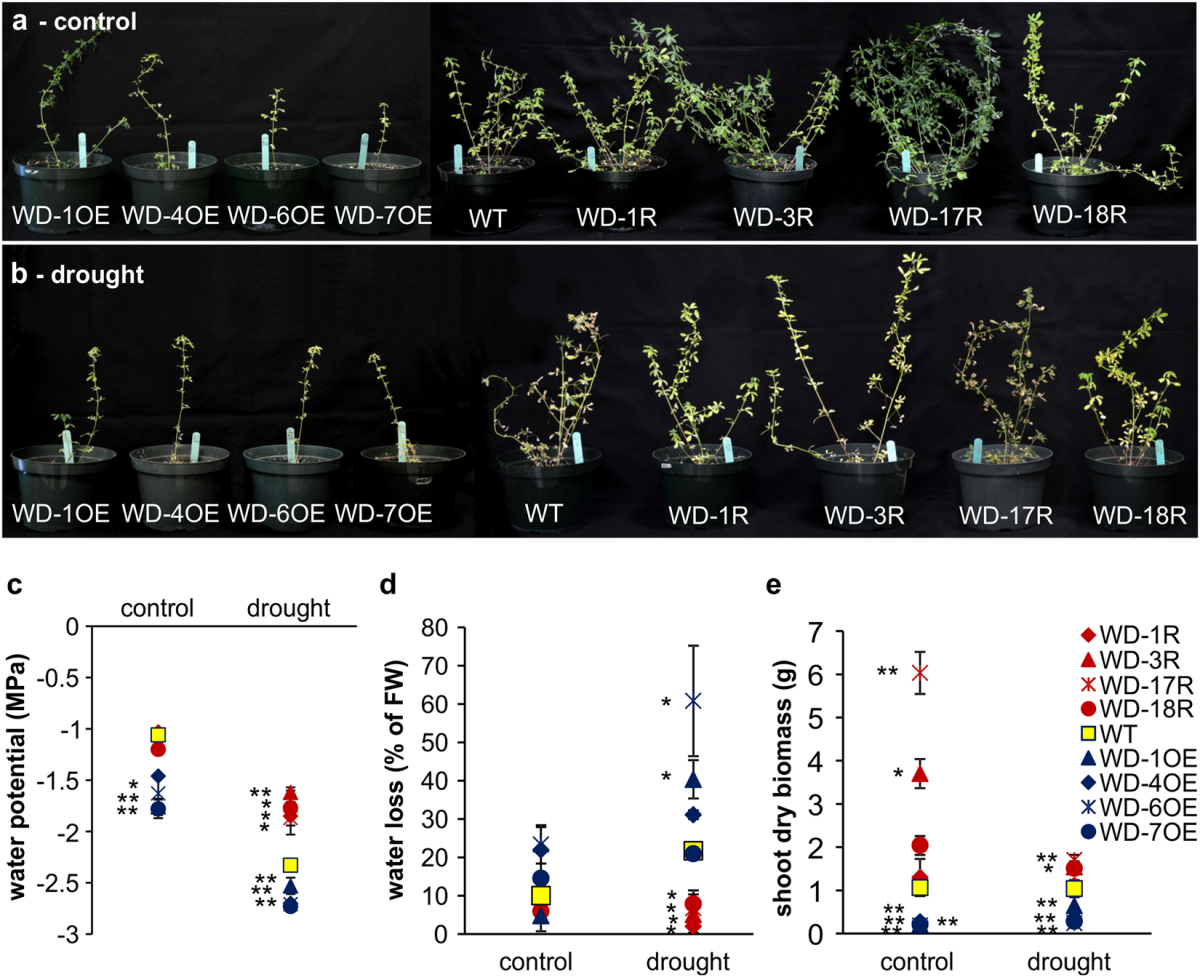
Effect of drought stress on water conservation and shoot biomass of alfalfa. WT and *WD40-2* overexpression, and RNAi genotypes (**a**) well-watered control conditions, (**b**) under drought stress, (**c**) leaf water potential, (**d**) water loss, and (**e**) shoot biomass. Data are average of 3-5 biological replications. Single asterisk (*) shows significance of *WD40-2* RNAi and overexpression plants with WT at P < 0.05 and double asterisk (**) indicates P < 0.01 (ANOVA).

Under non-stress control conditions, we observed stunted growth of transgenic alfalfa overexpressing *WD40-2*, where plants looked dwarf, small in stature with only one stem compared to WT (Fig. 7a). We noted that WD-6OE and WD-7OE plants (which have the highest *WD40-2* expression) exhibited the most severe phenotypes (Supplementary file S11; Fig. 7), suggesting high *WD40-2* expression leads to negative effects on plant growth. On the other hand, *WD40-2* RNAi genotypes looked large, green with multiple stems compared to WT (Fig. 7A). In addition, WD-3R and WD-17R with medium level of downregulation (Supplementary file S11) showed increased plant growth (Fig. 7a), indicating that reduced *WD40-2* expression at moderate levels enhances alfalfa performance.

We tested whether drought tolerance in alfalfa is affected by *WD40-2* expression. After withholding water for 22 days, *WD40-2* RNAi genotypes remained relatively green and turgid, but WT and *WD40-2* overexpression transgenic plants wilted (Fig. 7b). Moreover, *WD40-2* overexpression plants were dwarfed and wilted with a single stem compared to WT (Fig. 7b).

We also measured the changes in water potential and water loss with and without drought stress. We exposed wild-type, *WD40-2* overexpression and RNAi transgenic plants to drought by withholding water. Without drought, water potential in overexpression genotypes (except WD-4OE) was significantly reduced relative to WT while no water potential differences were found between WT and RNAi plants (Fig. 7c). During drought treatment, overexpression genotypes exhibited significantly lower, and RNAi genotypes significantly higher water potential than WT (Fig. 7c). These data suggest that drought causes a small reduction in water potential when *WD40-2* expression is reduced and vice versa.

We observed no differences of water loss among these plants under control conditions, water loss in response to drought was significantly increased in two *WD40-2* over-expression genotypes, and was significantly reduced in all *WD40-2* RNAi genotypes compared to WT (Fig. 7d). Thus, attenuated expression of *WD40-2* decreases water loss, making plants resistant to drought, suggesting that *WD40-2* plays an important role in controlling water status in alfalfa.

We compared the shoot biomass accumulation in WT and transgenic plants. The *WD40-2* overexpression plants showed a lower biomass than WT under non-stress. On the contrary, biomass in RNAi plants was significantly increased in two genotypes under this condition (Fig. 7e). Similarly, biomass accumulation was reduced in overexpression plants and increased in RNAi plants under drought stress (Fig. 7e). This result is consistent with the rapid wilting, lower water potential and faster water loss in overexpression plants, and higher water potential, reduced water loss and greenish plants of RNAi plants upon drought treatment.

### *WD40-2* affects root growth in alfalfa

**Figure 8:**
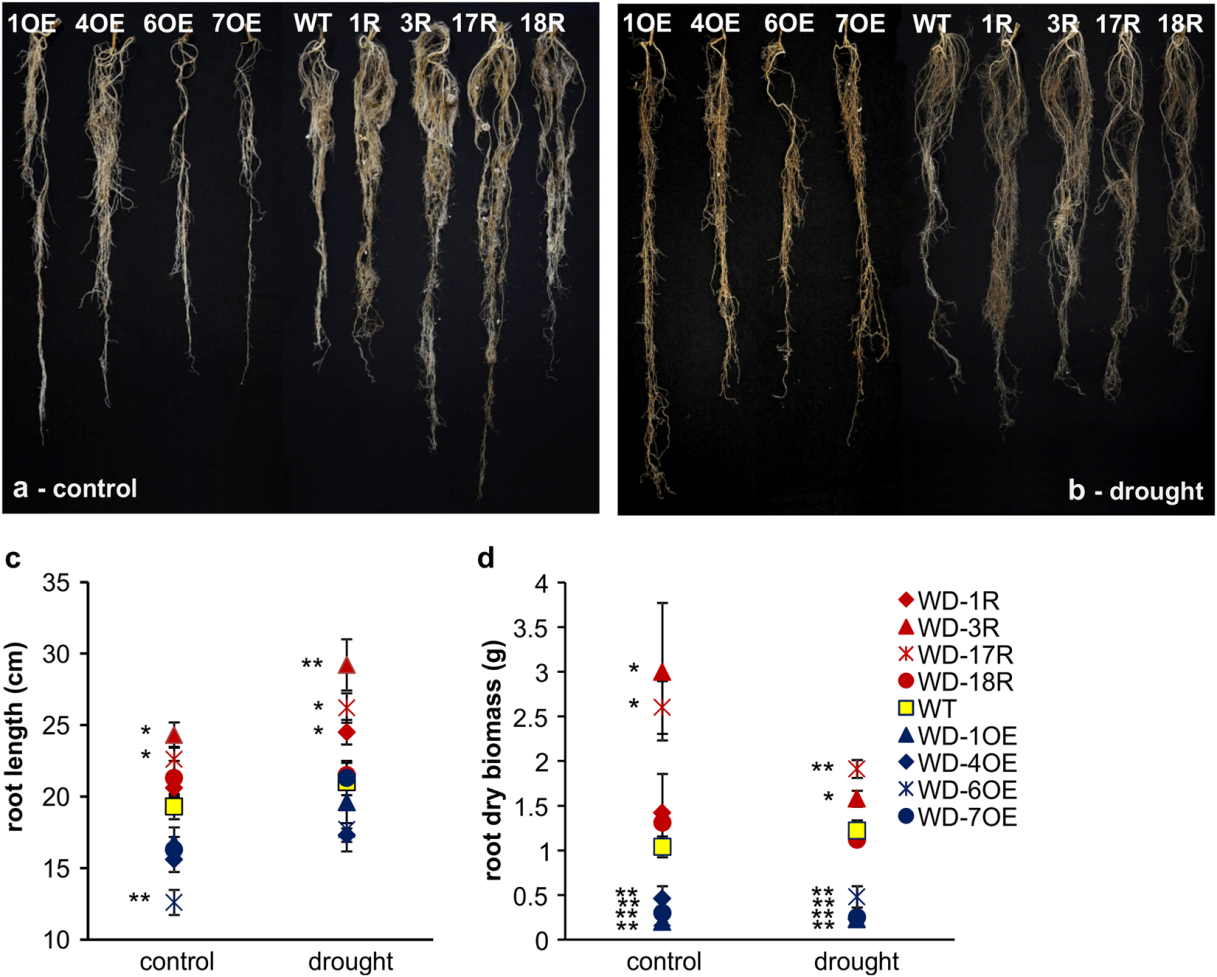
Effect of drought stress on root growth and root biomass of alfalfa. WT and *WD40-2* overexpression, and RNAi genotypes (**a**) well-watered control, (**b**) drought stress, (**c**) root length and (**d**) root biomass. Data are average of 3-5 biological replications. Single asterisk (*) shows significance of *WD40-2* RNAi and overexpression plants with WT at P < 0.05 and double asterisk (**) indicates P < 0.01 (ANOVA).

We analyzed differences in root length and biomass under non-stress control and drought stress conditions. Compared to WT, roots of over-expression genotypes looked thin and had a reduced volume, with one major root and only a few adventitious roots originating from the main root, during non-stress control conditions (Fig. 8a). Under identical conditions, thick, long roots with many adventitious roots were observed in RNAi genotypes compared to WT (Fig. 8a). Similar patterns were obtained during drought stress where roots of over-expression plants looked brownish and thin, whereas thick roots with extra adventitious roots were observed in RNAi plants compared to WT (Fig. 8b). We further quantified the root data and showed that without drought, one over-expression genotype (WD-6OE) had shorter roots than WT, whereas two RNAi genotypes (WD-3R, WD-17R) exhibited significantly increased root length relative to WT (Fig. 8c). During drought stress, overexpression plants did not show difference of root length with WT. RNAi plants, however, exhibited enhanced root length in three genotypes (Fig. 8c).

We further determined root biomass accumulation in WT and transgenic plants with increased or reduced *WD40-2* expression. All four overexpression genotypes showed significant reduction in root biomass than WT under control conditions whereas two RNAi genotypes (WD-3R, WD-17R) gained significantly higher root biomass than WT under this condition (Fig. 8d). Moreover, all over-expression genotypes accumulated lower biomass, while two RNAi genotypes built up more biomass than WT also under drought stress (Fig. 8d). Our root biomass data are consistent with root phenotype in over-expression and RNAi genotypes with and without stress (Fig. 8a,b).

## Discussion

Numerous studies have shown that miR156 affects biomass production and grain yield in plants^42–45^. Our group previously generated six alfalfa genotypes (A16, A8a, A8, A11, A17 and A11a) overexpressing miR156^25^. These alfalfa genotypes showed enhanced aerial biomass, root length, forage yield, quality and delayed flowering under non-stress conditions^25^. The increase in root length caused by miR156 suggested to us that miRNA may also be involved in controlling drought stress in alfalfa. Our current study revealed that two of the miR156OE genotypes A16b and A8 with moderate miR156 expression, also showed enhanced root length and, additionally showed reduced water loss under drought conditions. Previously, these two genotypes displayed the most pronounced phenotypes, such as increased root biomass, proline accumulation, relative water content and survival after drought stress^12^. We therefore chose them for RNA-Seq analysis. Transcriptomic analysis revealed that miR156 can affect: 1) differentially expressed genes commonly found in both genotypes 2), drought-specific genes commonly expressed in both genotypes and 3) genes expressed in an individual miR156OE genotype relative to non-stress WT control. Our study has found DEGs that are specifically found in miR156OE genotypes but not in WT under well-watered control (Supplementary file S13) and drought stress conditions (Supplementary file S14). These genes may provide an interesting insight on what processes, mechanisms and pathways are affected by miR156, and open an array for further studies.

Overall, GO-enrichment analysis revealed that miR156OE genotypes had a reduced number of genes that are downregulated under drought conditions in three functional categories; biological process, molecular function and cell component. The GO terms such as effect of ROS metabolic process, response to oxidative process, peroxidase activity and protein kinase activity are closely related to drought tolerant phenotypes^46^. Reduced number of downregulated genes in these categories may show that miR156 alleviates drought-related suppression of these genes for a better response to stress. On the other hand, we noted an increased number of upregulated genes in A16b in some functional categories such as carbohydrate metabolic process, secondary metabolic process, catalytic activity, cell periphery and plasma membrane. Higher levels of starch content in A16b compared to control and A8 plants^25^, may justify increased number of genes at least in one category “carbohydrate metabolic process” in A16b plants. In addition, increased number of upregulated genes in both genotypes (A16b, A8) belonging to the ‘response to abiotic stress stimuli’, may suggest a pronounced role for miR156 in drought response of alfalfa. Our group has previously shown that expression of several genes positively involved in abiotic stress responses was consistently upregulated in A16b and A8 under drought^12^, which supports the outcome of our current study.

Our results indicated many genotypic differences in DEGs in response to drought. During drought conditions, WT contained >400 DEGs compared to A16b and A8, which showed a similar number of DEGs under drought stress. Relative to the corresponding WT, numbers of DEGs in A16b were twice of those found in A8 under drought stress. This may indicate a stronger response by A16b to drought stress presumably due to different miR156 levels. A strong response of A16b was also observed in various GO categories such as “transport”, “carbohydrate metabolic process” “catalytic activity” “extracellular region” and “cell periphery”. These results showed that different levels of miR156 can affect similar genes and GO categories, as well as expression of some unique non-overlapping genes in each genotype^47^. For example, *CATALASE* and *ZEAXANTHIN EPOXIDASE* were upregulated in A16b, but not in A8, in response to drought. On the contrary, *GLUTATHIONE SYNTHETASE* expression was induced only in A8, but not in A16b under drought conditions^12^. These uniquely expressed genes in an individual genotype can bring about different degrees of phenotypic change in plants^13,47^.

Transcription factors (TFs) play a crucial role in regulating gene expression under abiotic stress in plants. The RNA-seq study revealed that expression of many TFs was altered in miR156OE genotypes under drought stress. Major TFs affected by drought stress in miR156OE genotypes belonged to bHLH, HD-ZIP, TCP, C2H2 and WRKY family. Previous studies have shown positive roles for these TFs in drought responses of various plant species^48–52^, indicating that miR156 improves drought responses in alfalfa by modulating expression of at least some members of these TF families. The *SQUAMOSA-PROMOTER BINDING PROTEIN-LIKE (SPL)* are known targets of miR156^25,47^, and the high number of *SPL*s that were downregulated in miR156OE genotypes under drought may indicates that at least some *SPL*s may act as negative regulators of drought responses. This is also evident from our previous study, which showed an improved drought tolerance in alfalfa genotypes with reduced expression of *SPL13*^12^. Moreover, we observed altered expression of *WD40* TFs in miR156 genotypes under drought stress. Given that *WD40* genes could be potential target of miR156^32^, we hypothesized that miR156 downregulates *WD40-2*, and decided to conduct functional characterization experiments with this non-SPL gene.

Our group has identified seven *SPL* genes that are targets of miR156 in alfalfa^25,47^. Given the diversity of important transcription factors, genes and physiological traits affected by *miR156* in alfalfa, it is critical to identify and characterize downstream target genes, not only *SPLs* but also other genes and transcription factors such as WD40. Genome wide analysis of the WD40 repeat proteins has been conducted in *Arabidopsis* and cucumber^53^, but their role has not been elucidated in alfalfa. In the present study, we focused on identifying non-SPL targets of miR156 and identified a gene encoding a putative WD40/repeat protein (*WD40-2*) that also regulates drought responses. Some studies have shown involvement of *WD40* in anthocyanin biosynthesis^33–36,54^, nodule development^38^, cell wall biosynthesis^37^, as well as hormone, light and stress responses^39^.

In addition to common miR156 targeted *SPL* genes in alfalfa^25,47^, *WD40-2* represents another family of genes targeted by miR156. Contrary to some *SPL* genes in *Arabidopsis* and alfalfa^25,55^, we did not detect cleavage sites of *WD40-2* within the predicted miR156 target region, but sites were instead located upstream of the predicted target region. This discrepancy could be due to an RNA-induced silencing complex affecting the transcript during miRNA-related cleavage^56^. Our results however are consistent with some previous studies that have also shown variation in cleavage sites, for example, a cleavage site of rice *SPL14* was found away from the miR156 target site^44^. In addition, our group has recently shown similar results where target sites of *SPL2*, *SPL3*, *SPL4* and *SPL9* were detected beyond the target sites^47^.

Two recent studies showed a positive role for *WD40* gene in drought response in *Arabidopsis*^57^, as well as salt and osmotic stress responses in wheat^58^. However, *Arabidopsis* and wheat *WD40* genes phylogenetically differ from alfalfa *WD40-2* (Supplementary file S12), indicating functional differences potentially among various species. Alfalfa plants overexpressing *WD40-2* exhibited stunted growth under control and drought stress conditions. In addition, low water potential, shoot, root biomass, and root length coupled with increased water loss further elucidates its role as a negative regulator. Furthermore, RNAi genotypes of *WD40-2* seem to alleviate the negative effects of drought, and show high water potential, increased shoot and root biomass along with root length, and reduced water loss under drought stress conditions. This provides evidence of *WD40-2* role as a negative regulator of drought response in alfalfa. In contrast, *WD40* has been shown to positively modulate osmotic stress responses in *Arabidopsis* and wheat^57,58^. Studies have shown that different genes belonging to the same family could have opposite roles in plants. For example, seven *Arabidopsis PP2C*s are negative regulators^59–65^, and one is a positive regulator of ABA signaling^66^. Recently, our group has discovered that one gene (*SPL13*) belonging to the miR156 network acts as a negative regulator of drought responses in alfalfa^12^. These observations support our current results which show that *WD40-2* negatively regulates drought responses in alfalfa through a relationship with *SPL13*.

In conclusion, we present here the first report on the effect of miR156 overexpression on global gene expression in alfalfa under drought stress. We identified a non-SPL gene (*WD40-2*) that is targeted by miR156. Functional assessment of *WD40-2* indicated that overexpressing and RNAi plants exhibited opposite effects, indicating its role as a negative regulator of drought responses in alfalfa. However, the possibility exists that the alfalfa drought tolerance phenotype is in part regulated by additional drought-responsive genes identified in our RNA-seq study, and these may directly or indirectly be regulated by miR156. For example, *SPL13* is directly regulated by miR156^25^, and downregulation of *SPL13* contributes to drought tolerance in alfalfa^12^. We have proposed a model that shows how miR156 regulates drought responses in alfalfa by targeting *WD40-2*, and affecting other physiological traits (Supplementary file S15). The model proposes that drought stimulates *miR156* expression, which in turn cleaves *WD40-2* transcript in alfalfa. Downregulation of *WD40-2* results in improved drought tolerance whereas overexpression negatively affects drought responses. This model suggests that *WD40-2* is a negative regulator of drought tolerance in alfalfa. Nonetheless, it is important to undertake functional and molecular studies also for at least some of DEGs under drought conditions. Similarly, a recent study shows that miR156 improves salinity stress tolerance in alfalfa^40^. Thus, a detailed analysis of the regulation of miR156 and downstream targeted drought-related genes remains an important study topic, as it will be of key interest to see whether miR156 and *WD40* gene family also play roles in improving tolerance to other stresses and whether they function similarly in other plant species.

## Methods

### Plant material, growth conditions and drought experiments

Alfalfa plants overexpressing microRNA156 (miR156OE) were developed previously by our group^25^ for use in this study. Rooted stem cuttings were made from two of these genotypes (A8 and A16b) and a wild type control genotype (WT) that was generated through plant tissue culture. Also, rooted stem cuttings were made from four *WD40-2* overexpression genotypes (WD40-1OE, WD40-4OE, WD40-6OE, WD40-7OE) and four RNAi genotypes (WD40-1R, WD40-3R, WD40-17R, WD40-18R). Stem cuttings were then transferred to pots (8 × 6′′) containing equal amount of homogenized PRO-MIX® BX soil. Plants were grown on a greenhouse bench under a 16-hour light/ 8-hour dark regime, and the soil was watered twice a week. Drought experiments were initiated on two-month-old plants as described in our previous study^12^. Briefly, at the start of each experiment, 50% soil moisture was established in each pot using a Fieldscout soil sensor (Spectrum Technologies Inc. Aurora, IL, USA). After commencing the drought experiment by withholding all water, soil pots were rotated randomly every day on the greenhouse bench to minimize environmental variation. Physiological data and root samples for RNA extraction were collected when WT plants showed stress symptoms i.e. wilting, drooping and brownish leaves, and soil moisture had dropped below 5% i.e. 13 days after imposing drought stress for miR156OE genotypes (A8, A16b), and 22 days for *WD40-2* overexpression and RNAi genotypes.

### Cloning *medicago sativa WD40-2*

Overexpression and RNAi constructs were constructed for the alfalfa homolog *WD40-2* gene (*MsWD40-2*) using the Gateway system (Thermo Fisher Scientific, Mississauga ON). For overexpression, the full-length homologue of the *Medtr2g028050* gene (a putative *M*. *truncatula* transducin/WD40 repeat gene) was amplified from *Medicago sativa* cDNA using primers with AttB sites, AttB1-WD40-2-cDNA and AttB2-WD40-2-cDNA (Supplementary Table 4), and cloned into the pDONR/Zeo entry vector. For RNAi, a 239 bp putative *WD40-2* fragment was amplified from *Medicago sativa* cDNA using additional primers with AttB sites, B1-WD2-RNAi and B2-WD2-RNAi (Supplementary Table 4), and cloned into the pDONR/Zeo entry vector. After PCR screening and validation by sequencing, LR reactions were performed for the overexpression and RNAi constructs to recombine the putative *WD40-2* fragments into the pMDC83 (overexpression) and pHELLSGATE12 (RNAi) vectors. Subsequently, overexpression and RNAi constructs were used to transform *Agrobacterium tumefaciens* strain EHA105, which was then used to transform alfalfa as described in our previous study^25^. QRT-PCR was used to detect transcript levels of *WD40-2* gene in *WD40-2* overexpressing and RNAi genotypes using primers WD2-qPCR-F and WD2-qPCR-R (Supplementary Table 4).

### Measurement of root length, water loss and water potential

For root length measurements, plants were removed from the pots and roots were fully cleaned of soil. Root length was then recorded by taking measurements with a scale from root neck to root tip of control and drought stressed alfalfa plants of WT and miR156OE and *WD40-2* overexpressing genotypes as well as *WD40-2* RNAi genotypes. To conduct a water loss assay, water was withheld on two-month-old plants growing in soil under greenhouse conditions for 12 days (WT and miR156OE genotypes) and 22 days (WT, *WD40-2* overexpressing, and RNAi genotypes), and water loss was measured as described previously^12,67^. After the water loss experiment, root and shoot dry weight was obtained by incubating samples at 65 °C for 5 days. Water potential was also measured on drought stressed and non-stressed controls plants of WT, *WD40-2* overexpression and RNAi genotypes using a Portable Plant Water Status Console (Soilmoisture Equipment Corp. Santa Barbara, CA, USA).

### Next generation sequencing (RNA-seq)

About 5 cm of drought stressed and control root tips (13 days after withholding water) were harvested from WT and miR156OE genotypes (A8, A16b), and immediately frozen in liquid nitrogen. High quality total RNA was extracted from roots following activated charcoal protocol^68^. The integrity of RNA samples was confirmed on an Agilent Bioanalyzer 2100 RNA Nano chip (Agilent Technologies). An RNA library was constructed and sequenced on an Illumina Hi-Seq. 2500 using paired-end 101 bp reads at the Centre for Applied Genomics (Sick Kids Hospital, Toronto, Canada) as a fee-for-service contract. Six biological replicates were sequenced for each WT and A16b (three control and three drought stressed) while seven biological replicates were sequenced for A8 (three control and four drought stressed).

### Analysis of differential gene expression

Raw Illumina pair-end reads were trimmed using Trimmomatic^69^ to obtain high quality reads (Q > 30). These high-quality reads were used to identify differentially expressed genes (DEGs). We also used the *M*. *trancatula* genome as a reference for alignment of RNA-Seq reads using Tophat (v2.0.10). Tophat output was then used for differential expression analysis using Cufflinks software^70^. Subsequently, differentially expressed genes were annotated and assigned to three major functional categories (biological process, molecular function, cell component) using the GO Term enrichment tool from PlantRegMap and the *M*. *truncatula* database at P ≤ 0.01^71^. Differentially expressed genes were also screened for transcription factor families using the Plant Transcription Factor Database - PlantTFDB; http://planttfdb.cbi.pku.edu.cn/^71,72^. Venn diagrams were generated using the Venny tool^73^.

### *De novo* assembly of transcriptome

Transcriptome *de novo* assembly of *Medicago sativa* was performed directly on the set of sequenced reads using the Trinity platform^41^. A pair end assembly was performed on each alfalfa genotype (WT, A8 and A16b). Parameters used for assembling the transcriptome are described in Supplementary file S1.

### Validation of RNA-Seq by quantitative real-time PCR

For qRT-PCR validation, RNA was treated with TURBO DNase (Ambion, Austin, TX). A total of 1 µg RNA was used to synthesize cDNA using an iScript cDNA synthesis kit (Bio-Rad Laboratories, Mississauga ON). qRT-PCR amplification was conducted using a C1000 Touch™ Thermocycler Real-Time PCR System (Bio-Rad, Canada) using SsoFast SYBR Green Master Mix (Bio-Rad Laboratories, Mississauga, ON). Alfalfa homologues for two well-known housekeeping genes, ubiquitin (*Medtr3g112230*) and elongation factor (*Medtr1g101870*) with little variation of expression in our RNA-seq study, were used as reference genes for qRT-PCR reactions. Gene-specific primers and primers for reference genes are listed in Supplementary Table 4.

### Detection of cleavage sites in WD40-2

Cleavage sites in alfalfa *WD40-2* genes were detected using 5′ rapid amplification of cDNA end (5′-RACE) as described by^25^. The experiment was conducted using a First Choice_RLM-RACE Kit (Ambion, Burlington, ON, Canada) according to the manufacturer’s instructions. PCR products from Inner 5′ RLM-RACE PCR were purified using a gel purification kit (Qiagen,Toronto, ON, Canada) and cloned into a pJET1.2/blunt cloning vector (Fermentas, Ottawa, ON, Canada). At least 25 clones were subjected to sequencing using a pJET1.2/blunt sequencing primer.

### Statistical analysis

GraphPad Prism software (https://www.graphpad.com/scientific-software/prism/) was used to statistically analyze the data. For comparisons between two groups the Student t-test was used whereas for means of more than two, an ANOVA was used followed by Tukey’s test for multiple comparisons.

## Electronic supplementary material

Supplementary Table 1

Supplementary Table 2

Supplementary Table 3

Supplementary Table 4

Supplementary file S1

Supplementary file S2.c1

Supplementary file S2.c2

Supplementary file S2.c3

Supplementary file S2.c4

Supplementary file S2.c5

Supplementary file S2.c6

Supplementary file S3

Supplementary file S4

Supplementary file S5

Supplementary file S6

Supplementary file S7

Supplementary file S8

Supplementary file S9

Supplementary file S10

Supplementary file S11

Supplementary file S12

Supplementary file S13

Supplementary file S14

Supplementary file S15
