# Supplementary material for: Transcriptome analysis of microRNA156 overexpression alfalfa roots under drought stress
Source: Sci Rep. 2018 Jun 19;8:9363. doi: 10.1038/s41598-018-27088-8 (PMC6008443; doi:10.1038/s41598-018-27088-8)
Supplement: Supplementary file 1 — Supplementary Table 1 [file 41598_2018_27088_MOESM1_ESM.pdf]

Title: Transcriptome analysis of microRNA156 overexpression alfalfa roots under drought stress

Authors: Muhammad Arshada, Margaret Y. Gruber, Abdelali Hannoufa

Supplementary Table 1: Total filtered reads used for RNA-seq data analysis in WT and miR156OE genotypes

**Sample**	**Total filtered reads**
WT-C	120,834,412
WT-D	120,964,828
A16b-C	124,750,168
A16b-D	117,043,884
A8-C	120,876,414
A8-D	151,058,912
