# Supplementary material for: Transcriptome analysis of microRNA156 overexpression alfalfa roots under drought stress
Source: Sci Rep. 2018 Jun 19;8:9363. doi: 10.1038/s41598-018-27088-8 (PMC6008443; doi:10.1038/s41598-018-27088-8)
Supplement: Supplementary file 2 — Supplementary Table 2 [file 41598_2018_27088_MOESM2_ESM.pdf]

Title: Transcriptome analysis of microRNA156 overexpression alfalfa roots under drought stress

Authors: Muhammad Arshada, Margaret Y. Gruber, Abdelali Hannoufa

Supplementary Table 2: Validation of RNA-seq data by qRT-PCR. Data show gene expression in WT and miR156OE genotypes under drought conditions relative to corresponding WT and miR156OE well-watered control plants.

**WT**			**A16b**			**A8**		
**Gene ID**	**RNA-seq**	**qRT-PCR**	**Gene ID**	**RNA-seq**	**qRT-PCR**	**Gene ID**	**RNA-seq**	**qRT-PCR**
Medtr4g083570	-4.60	-4.87	Medtr4g017200	-5.23	-5.95	Medtr7g087070	-4.00	-1.60
Medtr1g115270	-3.83	-5.31	Medtr1g090667	-3.03	-6.82	Medtr1g090667	-3.76	-1.44
Medtr4g133800	-2.44	-4.77	Medtr4g083570	-2.00	-2.46	Medtr4g015450	-1.77	-1.26
Medtr4g026030	-1.60	-2.24	Medtr3g085700	0.14	0.11	Medtr4g017200	-1.24	-1.02
Medtr3g081580	0.22	0.35	Medtr8g107250	0.86	0.23	Medtr5g063080	0.18	0.86
Medtr2g065550	0.41	1.11	Medtr3g081580	1.82	1.92	Medtr7g111380	1.2	2.46
Medtr2g044140	1.06	1.66	Medtr2g044140	2.61	1.38	Medtr1g105615	3.12	2.85
Medtr1g102510	2.03	1.70	Medtr2g065550	3.11	3.84	Medtr2g070460	3.43	2.58
Medtr4g011250	4.83	3.00				Medtr2g049020	4.90	1.14
Medtr4g099370	8.41	7.96						
