# Supplementary material for: Transcriptome analysis of microRNA156 overexpression alfalfa roots under drought stress
Source: Sci Rep. 2018 Jun 19;8:9363. doi: 10.1038/s41598-018-27088-8 (PMC6008443; doi:10.1038/s41598-018-27088-8)
Supplement: Supplementary file 3 — Supplementary Table 3 [file 41598_2018_27088_MOESM3_ESM.pdf]

Title: Transcriptome analysis of microRNA156 overexpression alfalfa roots under drought stress

Authors: Muhammad Arshada, Margaret Y. Gruber, Abdelali Hannoufa

Supplementary Table 3: Transcription factors differentially expressed in miR156OE genotypes relagtive to WT

Transcription factor family	drought				control			
	**A16b**		**A16b**		**A16b**		**A8**	
	Up	Down	Up	Down	Up	Down	Up	Down
MYB	6	6	9	6	6	8	4	7
TCP	4	6	0	9	6	8	9	9
bHLH	6	18	2	11	3	12	6	15
BES1	0	0	3	3	0	1	0	3
bZIP	6	6	0	10	1	0	1	10
C2H2	3	0	4	1	1	0	1	1
AP2	1	0	1	0	1	0	1	1
HD-ZIP	3	3	3	4	1	6	3	4
NAC	1	9	0	3	1	3	0	4
WRKY	1	2	0	0	0	14	10	2
GRAS	1	0	1	0	1	0	0	1
SBP	1	2	1	4	0	0	0	0
WD40	3	3	2	2	3	2	1	3
Others	14	13	7	6	8	3	12	8
