# Supplementary material for: Transcriptome analysis of microRNA156 overexpression alfalfa roots under drought stress
Source: Sci Rep. 2018 Jun 19;8:9363. doi: 10.1038/s41598-018-27088-8 (PMC6008443; doi:10.1038/s41598-018-27088-8)
Supplement: Supplementary file 4 — Supplementary Table 4 [file 41598_2018_27088_MOESM4_ESM.pdf]

**Title:** Transcriptome analysis of microRNA156 overexpression alfalfa roots under drought stress

**Authors:** Muhammad Arshada, Margaret Y. Gruber, Abdelali Hannoufa

**Supplementary Table 4. Primers**

| Primer name | Primer Seq |
|---|---|
| AttB1-WD40-2-cDNA | GGGGACAAGTTTGTACAAAAAAGCAGGCTTCGCATCCTTATCCCTGAG |
| AttB2-WD40-2-cDNA | GGGGACCACTTTGTACAAGAAAGCTGGGTCTGAGCCATGTCAGTTCACCT |
| B1-WD2-RNAi | GGGGACAAGTTTGTACAAAAAAGCAGGCTTC GCCACTGGGAACCAAGATAA |
| B2-WD2-RNAi | GGGGACCACTTTGTACAAGAAAGCTGGGTC GGGCTAAAAGATACGCCTGA |
| WD2-qPCR-F | GGGATGTTCGGAATCTTTCA |
| WD2-qPCR-R | CAAATCCTTGCTTTGCATCA |
| WD2-seq-F | GGGATGTTCGGAATCTTTCA |
| WD2-seq-R | CAAATCCTTGCTTTGCATCA |
| Medtr1g090667-F | GTGATTGTGGGAGCTGGAAT |
| Medtr1g090667-R | TCATAGGGTCCATGCTTTCC |
| Medtr7g087070-F | GTTGTCCCAAAGGAGTGCAT |
| Medtr7g087070-R | GCCCATGTATGCTCCAGACT |
| Medtr3g085700-F | ATCTATTCCACCACCGATGC |
| Medtr3g085700-R | TCTCTCCGTCGACAGATCCT |
| Medtr7g111380-F | TCCTTCCACCTCTTGGTGTC |
| Medtr7g111380-R | CGAGGGGGTAATTGGAAGAT |
| Medtr1g105615-F | CGAATTGCAACCCCTCTAAA |
| Medtr1g105615-R | ATACCCATTGTGGCTGCTTC |
| Medtr2g049020-F | CGATCGATTTGGAAGGAAAA |
| Medtr2g049020-R | TCCAACACCAAGACCAACAA |
| Medtr4g017200-F | GGCTTGCTTTCTTTCAGTGC |
| Medtr4g017200-R | TGGTTTGCCTGATTTTAGCC |
| Medtr4g015450-F | TTGTTGTTGCTCCCACCATA |
| Medtr4g015450-R | TTACATGCGCGAATTTCTTG |
| Medtr5g063080-F | TTGTTTGGTGGGCTTTTAGG |
| Medtr5g063080-R | TCCAATGAGTTTGGTCACGA |
| Medtr4g083570-F | TGAAAGTGACGAGCCACAAC |
| Medtr4g083570-R | CATTTGATGGAAGCACATGG |
| Medtr8g107250-F | CCGGGAATCTTAAGCAACAA |
| Medtr8g107250-R | GCCGATGAGTGTATGGTCCT |
| Medtr3g081580-F | CAAGTGCAATCGGTGTTTGT |
| Medtr3g081580-R | CAAAAGCATCAGGAAGCACA |
| Medtr2g044140-F | CTTGGTGCATGGAAATGTTG |
| Medtr2g044140-R | TACCCAATGACCACGGAAAT |
| Medtr2g065550-F | CAATCTCGTTCCAGCAGTGA |
| Medtr2g065550-R | TGTGCATTTAGCAGCTTTGG |
| Medtr1g115270-F | TGAAACACATGCCCACATCT |
| Medtr1g115270-R | ATTCCATCCTTACCGGCAGT |

| | |
|---|---|
| Medtr4g133800-F | TACCACCTCCTGAAGCCTTG |
| Medtr4g133800-R | TAACGGCACAATCATGGAAA |
| Medtr4g026030-F | TGCTTGGGCTATTCAGAGAGA |
| Medtr4g026030-R | CGACCAGCTCCAAATGGTAT |
| Medtr1g102510-F | TGTCAAGGAAGCAACCAACA |
| Medtr1g102510-R | AGAAGCATCCAAAAGCTTGC |
| Medtr4g011250-F | GAGACCAAGGGTCGAACTGA |
| Medtr4g011250-R | GGGCTGTCTGTCCTGTCTTC |
| Medtr4g099370-F | GAATGATGGCAATGTTGCTG |
| Medtr4g099370-R | CCACTACGGTTGCTCCATTT |
| Medtr7g102450-F | GTGCAACAGGACCAACACAC |
| Medtr7g102450-R | GATGAAGGGGTTTGCTGAGA |
| Medtr4g099370-F | GAATGATGGCAATGTTGCTG |
| Medtr4g099370-R | CCACTACGGTTGCTCCATTT |
| Medtr6g088785-F | GCTAAAAGGGCCACTTCCAT |
| Medtr6g088785-R | TATTTCGCCGGAGAATTGAT |
| Medtr4g094428-F | GACCTGGTGGGTGTGAGTTT |
| Medtr4g094428-R | CACTCTCACGTTGCAGTGGT |
| Medtr3g072560-F | ACAGTTGTGTGGGGAAAAGC |
| Medtr3g072560-R | TTTCATCCTTTGGTGGTGGT |
| Medtr3g082770-F | TGTGGATCAAATTGCCAAGA |
| Medtr3g082770-R | GAAGCATTTGCAGGAATGGT |
| Medtr8g096730-F | AGTTGTTTCCACGACCTGCT |
| Medtr8g096730-R | TTCCGGTGGAGAAAGTATGG |
| Medtr5g082150-F | TTCTTTCCACCGCGTAAATC |
| Medtr5g082150-R | ATCGGAAACCACGAGAACAG |
| Medtr3g108240-F | TAATGCACTCCGAGCCTCTT |
| Medtr3g108240-R | TGTGGTCTGGTGATTGGCTA |
| Medtr5g070330-F | TCATCTCGGTTGCATTACCA |
| Medtr5g070330-R | AGGCCTTGGATTTTTGCTTT |
| Medtr2g070070-F | TGGAGGAAAATGTGGCTTTC |
| Medtr2g070070-R | AGTGGCACCACCGTAAAAAG |
| Medtr2g015720-F | TGTGGGAATCACTGTCGTGT |
| Medtr2g015720-R | CGTCCAACGTTTTCTCGAAT |
| Medtr5g030130-F | AGATGTCCCCACAATCTTGC |
| Medtr5g030130-R | TTGAATGAAAGGCCTCAACC |
| Medtr5g054900-F | AGGTCAGCCTTGTGAAGGAA |
| Medtr5g054900-R | TCACGGCATGGAAGTATTGA |
| Medtr2g073420-F | GAGAATGGCCAAAAGATGGA |
| Medtr2g073420-R | GCTTTTGACATTGCATGTGG |
| Medtr2g070460-F | GGAACACGAGAAGGAAGCTG |
| Medtr2g070460-R | GGGGAGCCTCTGTTTTTACC |
| Medtr7g045860-F | CCACCGACAATTGGAAACTT |
| Medtr7g045860-R | CCAATGCAAATGTTGTGAGG |

| | |
|---|---|
| Medtr0052s0160-F | CCGTGAACCATCGAGTTTTT |
| Medtr0052s0160-R | CCCTGCACGCAATACCTAAT |
| Medtr4g045990-F | GAAGAAAGCTTCTTTGCTTGAGA |
| Medtr4g045990-R | TGGCAGCTAGTGTTGGTGTC |
| Medtr6g011490-F | GCCTGTTGTTGTTCATGTGG |
| Medtr6g011490-R | TGAACCCTCAAAGTCCCAAG |
| Medtr3g112230-F | CACCTACAACCCGCAATCTT |
| Medtr3g112230-R | TTTCAGACCCAGGATTCGAC |
| Medtr1g101870-F | CCAACTGGACTGCAAACTGA |
| Medtr1g101870-R | CACGCTTGAGATCCTTGACA |
