# Supplementary material for: Transcriptome analysis of microRNA156 overexpression alfalfa roots under drought stress
Source: Sci Rep. 2018 Jun 19;8:9363. doi: 10.1038/s41598-018-27088-8 (PMC6008443; doi:10.1038/s41598-018-27088-8)
Supplement: Supplementary file 5 — Supplementary file S1 [file 41598_2018_27088_MOESM5_ESM.pdf]

Title: Transcriptome analysis of microRNA156 overexpression alfalfa roots under drought stress

Authors: Muhammad Arshad, Margaret Y. Gruber, Abdelali Hannoufa

Supplementary file S1:

Parameters for RNA-Seq data analysis using tophat and cufflinks

1. Map the reads for each sample to the reference genome

tophat -p 8 -G Medicago_truncatula.MedtrA17_4.0.31.gtf -o th_out genome WT_C1_R1.fastq WT_C1_R2.fastq

2. Assemble transcripts for each sample with reference gene structure

cufflinks -p 8 –o cuff_out th_out/accepted_hits.bam

3. Run Cuffmerge on all assemblies to create a single merged transcriptome annotation:

cuffmerge –g Medicago_truncatula.MedtrA17_4.0.31.gtf –s Mt.fa –p WT.txt

4. Run Cuffdiff by using the merged transcriptome assembly along with the BAM from Tophat for each replicate

cuffdiff -o diff_out -b Mt.fa -p 8 –L WT,miR156OE -u merged_asm/merged.gtf ./WT_tophat1/accepted_hits.bam ./WT_tophat2/accepted_hits.bam

Parameters used for assembling *M.sativa* transcriptome

./Trinity.pl --seqType fq --left reads.ALL.left.fq --right reads.ALL.right.fq --CPU 8 --max_memory 500G

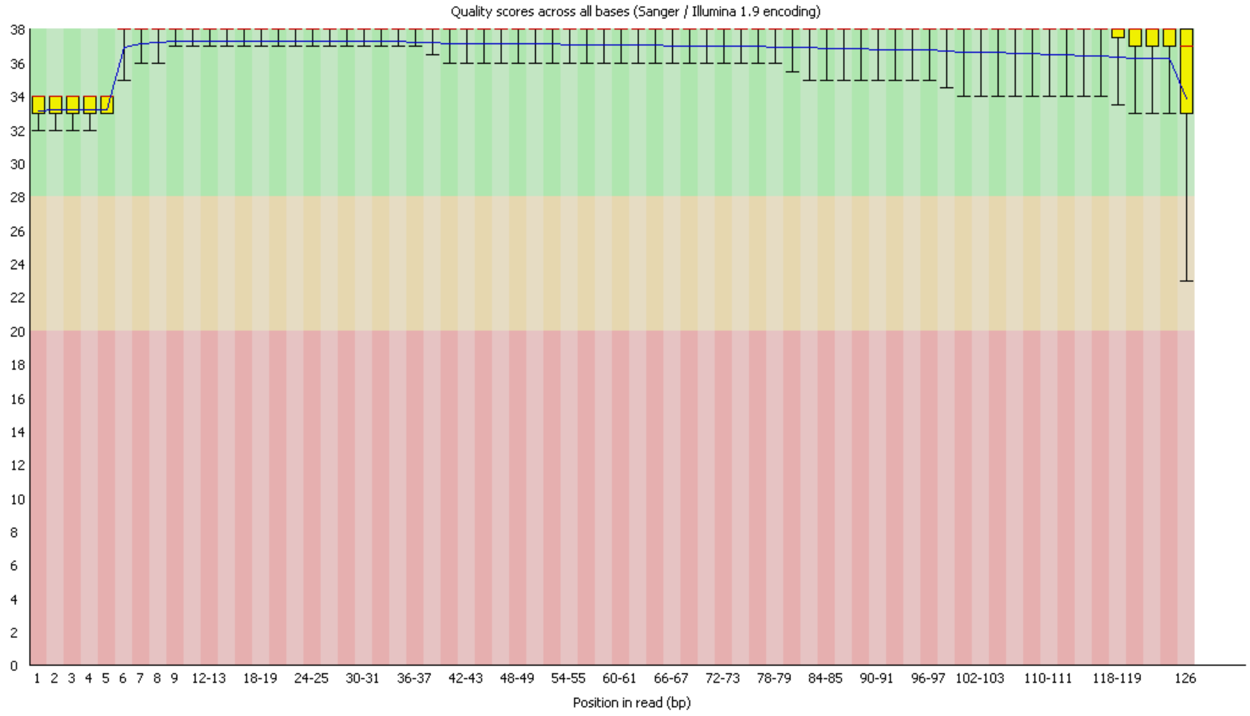

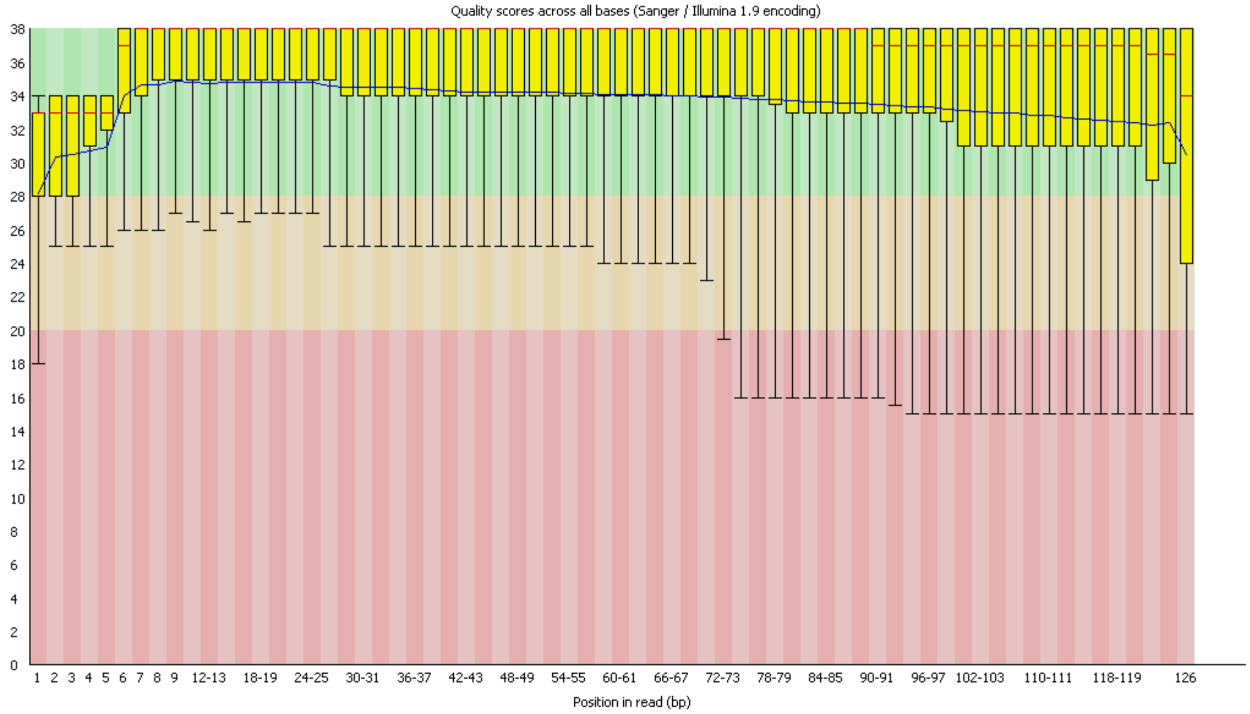

RNA-Seq raw reads QC results (per-base quality)
