# Supplementary material for: Transcriptome analysis of microRNA156 overexpression alfalfa roots under drought stress
Source: Sci Rep. 2018 Jun 19;8:9363. doi: 10.1038/s41598-018-27088-8 (PMC6008443; doi:10.1038/s41598-018-27088-8)
Supplement: Supplementary file 19 — Supplementary file S10 [file 41598_2018_27088_MOESM19_ESM.pdf]

Transcriptome analysis of microRNA156 overexpression alfalfa roots under drought stress

Muhammad Arshad, Margaret Y. Gruber, Abdelali Hannoufa

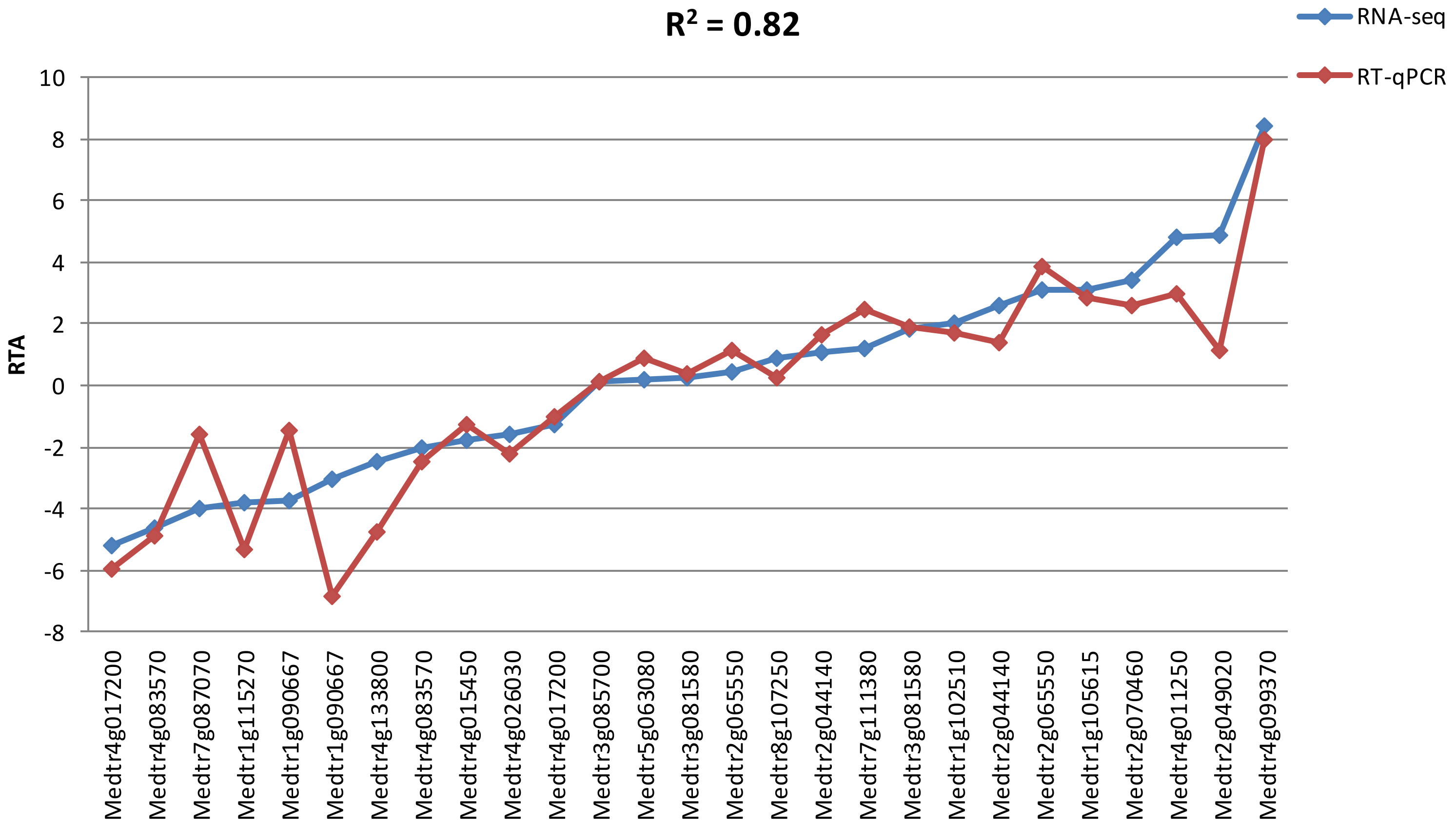

Supplementary file S10: Correlation between RNA-seq and RT-qPCR results.
