# Supplementary material for: Transcriptome analysis of microRNA156 overexpression alfalfa roots under drought stress
Source: Sci Rep. 2018 Jun 19;8:9363. doi: 10.1038/s41598-018-27088-8 (PMC6008443; doi:10.1038/s41598-018-27088-8)
Supplement: Supplementary file 20 — Supplementary file S11 [file 41598_2018_27088_MOESM20_ESM.pdf]

Transcriptome analysis of microRNA156 overexpression alfalfa roots under drought stress

Muhammad Arshad, Margaret Y. Gruber, Abdelali Hannoufa

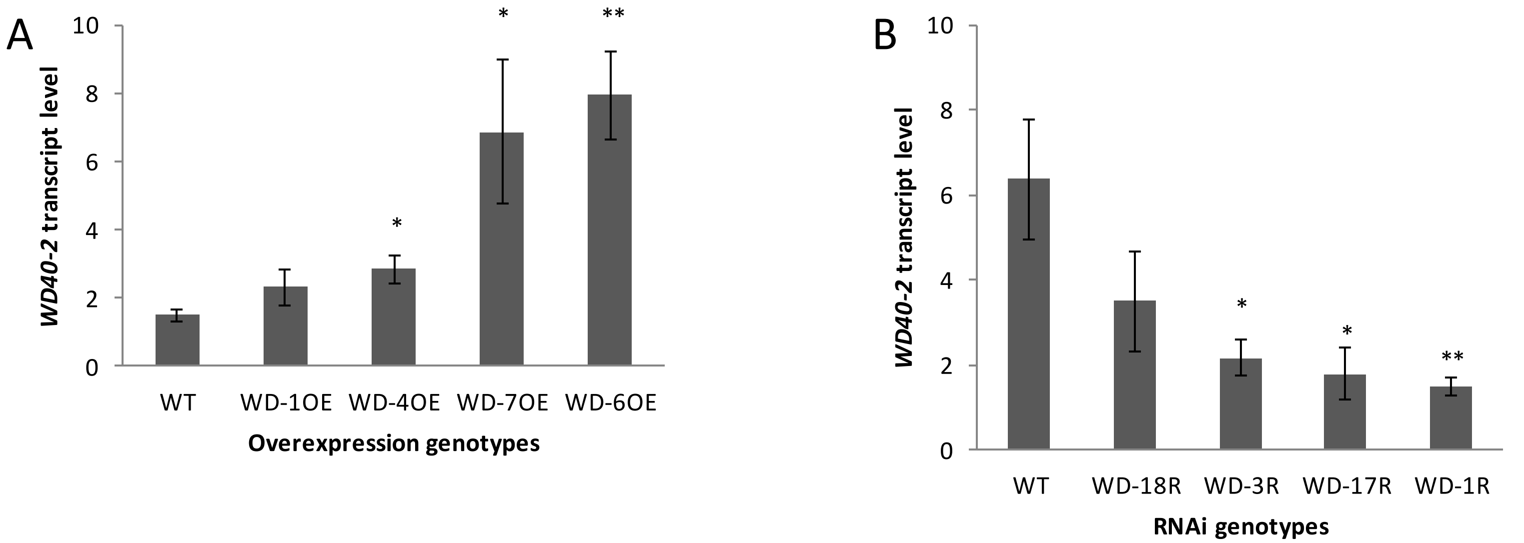

Supplementary file S11: Expression analysis of *WD40-2* in WT and *WD40-2* (**A**) overexpression, and (**B**) RNAi genotypes. Data are average of four biological replications. Single asterisk (*) shows significance of *WD40-2* RNAi and overexpression plants with WT at $P < 0.05$ and double asterisk (**) indicates $P < 0.01$ (t-test).
