# Supplementary material for: Transcriptome analysis of microRNA156 overexpression alfalfa roots under drought stress
Source: Sci Rep. 2018 Jun 19;8:9363. doi: 10.1038/s41598-018-27088-8 (PMC6008443; doi:10.1038/s41598-018-27088-8)
Supplement: Supplementary file 24 — Supplementary file S15 [file 41598_2018_27088_MOESM24_ESM.pdf]

Transcriptome analysis of microRNA156 overexpression alfalfa roots under drought stress

Muhammad Arshad, Margaret Y. Gruber, Abdelali Hannoufa

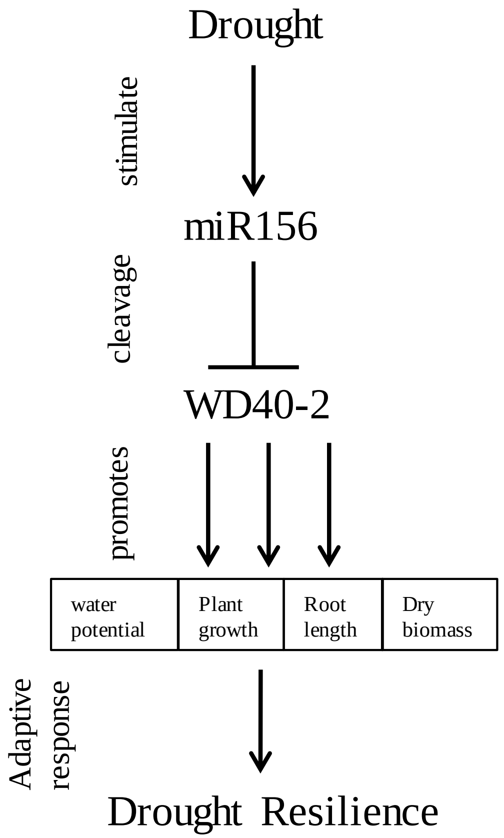

Supplementary figure S15: A pathway showing the role of miR156 and WD40-2 in mediating drought responses in alfalfa. Drought stimulates miR156 expression. MiR156 then targets and downregulates WD40-2, which in turn improves drought tolerance by enhancing root growth and biomass, water potential and reducing water loss.
